# Orthostatic Intolerance after COVID-19 Infection: Is Disturbed Microcirculation of the Vasa Vasorum of Capacitance Vessels the Primary Defect?

**DOI:** 10.3390/medicina58121807

**Published:** 2022-12-08

**Authors:** Klaus J. Wirth, Matthias Löhn

**Affiliations:** Institute of General Pharmacology and Toxicology, University Hospital Frankfurt am Main, Goethe-University, Theodor-Stern Kai 7, D-60590 Frankfurt, Germany

**Keywords:** orthostatic intolerance, orthostatic dysfunction, cerebral blood flow, COVID-19, myalgic encephalomyelitis/chronic fatigue, capacitance vessels, vasa vasorum, microcirculation, Long COVID

## Abstract

Following COVID-19 infection, a substantial proportion of patients suffer from persistent symptoms known as Long COVID. Among the main symptoms are fatigue, cognitive dysfunction, muscle weakness and orthostatic intolerance (OI). These symptoms also occur in myalgic encephalomyelitis/chronic fatigue (ME/CFS). OI is highly prevalent in ME/CFS and develops early during or after acute COVID-19 infection. The causes for OI are unknown and autonomic dysfunction is hypothetically assumed to be the primary cause, presumably as a consequence of neuroinflammation. Here, we propose an alternative, primary vascular mechanism as the underlying cause of OI in Long COVID. We assume that the capacitance vessel system, which plays a key role in physiologic orthostatic regulation, becomes dysfunctional due to a disturbance of the microvessels and the vasa vasorum, which supply large parts of the wall of those large vessels. We assume that the known microcirculatory disturbance found after COVID-19 infection, resulting from endothelial dysfunction, microthrombus formation and rheological disturbances of blood cells (altered deformability), also affects the vasa vasorum to impair the function of the capacitance vessels. In an attempt to compensate for the vascular deficit, sympathetic activity overshoots to further worsen OI, resulting in a vicious circle that maintains OI. The resulting orthostatic stress, in turn, plays a key role in autonomic dysfunction and the pathophysiology of ME/CFS.

## 1. Introduction

Following COVID-19 infection, a substantial fraction of patients suffer from persistent symptoms known as Long COVID [1,2,3,4,5,6,7,8]. Long-lasting and exhausting fatigue, Post-Exertional Malaise (PEM), cognitive dysfunction, dyspnea and signs and symptoms of orthostatic intolerance (OI) are among the main symptoms [9,10,11,12,13,14]. These are also core symptoms of myalgic encephalomyelitis/chronic fatigue syndrome (ME/CFS) [15]. OI is highly prevalent in ME/CFS [16,17,18] and also occurs early in Long COVID [9]. Upon assuming the upright position, patients experience a worsening of symptoms and a decrease in cerebral blood flow occurs, while hypotension and orthostatic tachycardia are less consistent findings. The causes for the development of OI following acute COVID-19 infection are unknown. Since a portion of Long COVID patients develop a disease indistinguishable from ME/CFS [8,9], investigation into the causes of the early occurrence of OI after COVID-19 infection may also give clues to the understanding of the pathophysiology of OI in ME/CFS or ME/CFS itself.

It is commonly believed that autonomic dysfunction is the primary cause of orthostatic dysfunction (OD) [9,19]. Here, we present a different explanation for the pathogenesis of OI that considers the role of the blood vessel system, namely the capacitance vessels, in orthostatic regulation (OR). We do not question that autonomic dysfunction is also present [20,21,22,23,24,25] and contributes to the pathophysiology of OI. However, we think that it is not the primary cause but rather develops secondarily as a consequence of an underlying vascular disturbance caused by the COVID-19 infection.

## 2. Orthostatic Intolerance after COVID-19 Infection: Is Disturbed Microcirculation of the Vasa Vasorum of Capacitance Vessels the Primary Defect?

Before discussing in detail how vascular dysfunction could cause and explain OD in Long COVID, we first describe four main mechanisms that are involved in physiological and pathophysiological OR.

### 2.1. Impact of Capacitance Vessels and Autonomic Regulation on OR

During OR, large arterial and venous blood vessels contract to maintain the circulating blood volume and blood pressure. Capacitance vessels below the heart that have been distended by the sudden elevated hydrostatic pressure after a change from the lying into the upright position contract to counteract distension that otherwise would reduce the circulating blood volume and preload to the heart. Capacitance vessels above the heart contract to compensate for the distension-induced volume loss in the periphery. This function implies that these vessels relax at rest to take up blood that can be delivered into the systemic circulation during OR and exercise. Adaptation to exercise (ergoreflex and metaboreflex) requires an even higher degree of contraction of those vessels together with a diversion of blood from the intestines and other organs to the skeletal muscles, the heart and the brain. From a theoretical point of view, the capacitance vessels system can be impaired by several, even opposing disturbances.

(1) Capacitance vessels do not adequately contract when needed (behaving like a “flaccid tube system”) due to a failure to contract the smooth muscles located in the media of the vessel wall.

(2) Capacitance vessels may structurally be rigid and additionally shrunk, therefore being unable to dilate and to take up blood at rest, which then cannot be delivered in sufficient quantity into the systemic circulation during OR (a rigid and perhaps shrunk tube system).

(3) Inappropriate filling of the vascular system, or hypovolemia, causes OI and is consistently found in ME/CFS with low renin (renin paradox) [26]. A hypothesis for this paradox is given elsewhere [27].

(4) Inappropriate autonomic regulation (baroreflex, volume regulation, volume- and barosensors and vagal and sympathetic activity) occurs if autonomic OR is too weak, too slow or if it is exaggerated. Exaggerated sympathetic activity during OR is supposed to cause cerebral vasoconstriction. Apart from hypovolemia, autonomic dysfunction is clearly present in ME/CFS and Long COVID. Hyperventilation, which is also reported to occur in ME/CFS and Long COVID, may contribute to cerebral vasoconstriction [18].

### 2.2. Disturbed Microcirculation as an Alternative Trigger for a Dysfunctional OR?

A severe vascular disturbance occurs in COVID-19 infection and persists to a certain level in Long COVID, and accounts for the high vascular event rate during and after COVID-19 infection [28]. Several mechanisms have been reported to perturb the microcirculation, like endothelial dysfunction, formation of microthrombi and altered rheological properties of blood cells [29,30,31,32,33,34]. Signs of autonomic dysfunction are also found in Long COVID [20,21,22,23,24,25], but we question that autonomic dysfunction is the primary cause of OI.

Disturbed microcirculation mainly affects blood flow in small vessels and capillaries. Large and medium sized vessels are also perfused and nourished by small vessels; the vasa vasorum, that supply the adventitia and large part of the media of the vessel wall [35,36,37,38,39]. Large arteries of the systemic circulatory system, the pulmonary arteries and particularly large and medium sized veins are endowed with vasa vasorum. The veins being the most important capacitance vessels are endowed with extensive vasa vasorum, because of the low luminal (venous) partial oxygen pressure, which does not enable sufficient oxygen supply from the lumen to the vessel wall, in contrast to arteries [40,41]. Since these veins have an active role in OR and since venous oxygen partial pressure is most likely insufficient to fully support their active contractile function, they are critically dependent on arterial blood supply from their vasa vasorum. The latter are functional endarteries receiving no collateral blood flow from neighbouring microvessels [36] so that a disturbed perfusion or even an obstruction of such a supplying small vessel will cause hypoxia, which then can impair contractile function and also affect structure. Without appropriate contractile response of the capacitance vessels, orthostatic dysregulation occurs when assuming the upright position. Several papers report that the vasa vasorum of large vessels are affected after COVID-19 [35,36,37,38,39]. It has already been postulated that the involvement of the large vessels during COVID-19 infection found both in children and in adults is likely due to dysfunction of their vasa vasorum, and SARS-CoV-2 induced microthrombosis of vasa vasorum would lead to hypoxic conditions in the adventitia [36]. More systematic investigation is certainly needed to substantiate these findings made in small studies so far.

Smooth muscle cells in the media of capacitance vessels actively contract when being stimulated by alpha-adrenergic receptors during OR and for adaptation of the cardiovascular system to exercise (raising preload of the heart). Inflammation, malnutrition and hypoxia, as a consequence of disturbed microcirculation, may affect the contractile function of the smooth muscle cells to cause not only OD but also maladaptation of the cardiovascular system to exercise by the inability to raise cardiac preload and, thereby, cardiac output. Aortic inflammation was found after COVID-19 infection with 18-Fluorodeoxyglucose investigation [42]. This, together with the microcirculatory disturbance in skeletal muscle itself, may affect muscular perfusion to cause fatigue. Skeletal muscle hypoperfusion, as a result of those vascular disturbances, together with mitochondrial dysfunction in skeletal muscle, which we assume is due to an ionic disturbance of sodium and calcium handling, could explain the high fatigability, loss of force and skeletal muscle complaints like myalgia [43,44]. A wide variety of histological changes including mitochondrial alterations, inflammation and capillary injury were found in muscle biopsies of patients with post-COVID-19 complaints [45].

Being unable to adequately contract, the vascular capacitance system would behave as a “flaccid tube system.” An expected consequence of this vascular failure would be a rise in sympathetic activity to compensate for the deficit. An appropriate compensation will not always be possible, sympathetic activity is expected to strongly rise and to finally overshoot. Beyond a certain level, sympathetic activation becomes counterproductive and harmful by itself, thereby worsening OD by causing excessive, mainly cerebral and skeletal muscle alpha-adrenergic receptor mediated vasoconstriction, which has the potential of causing a vicious circle and of fixing OI so that it persists. In ME/CFS, reduced cerebral blood flow was even found in a sitting position, and in severe ME/CFS, even at 20 degrees of head-up tilt [46,47]. This means that in the awake state in human everyday life, orthostatic stress is almost unavoidable in ME/CFS. Chronic orthostatic stress has the potential to desensitize β 2-adrenergic receptors, which are very important in skeletal muscle physiology, and also the alpha2-adrenergic (inhibitory) autoreceptors, whose dysfunction could cause sympathetic and adrenergic hyperactivity and hypervigilance, as described previously [44,48]. Another potential disturbance should be considered as an additional vascular mechanism contributing to OD. In Long COVID, autoantibodies have been found targeting β2- and adrenergic α1-receptors, the MAS-receptor and the angiotensin-II-type-1 receptor. The presence of these autoantibodies could be linked to an impaired retinal capillary microcirculation, potentially mirroring the systemic microcirculation [49]. Such autoantibodies against vascular regulators have the potential to disturb OR as a highly coordinated vascular process. This is another argument for the idea of a vascular disturbance as the cause of OI.

**Figure 1:**
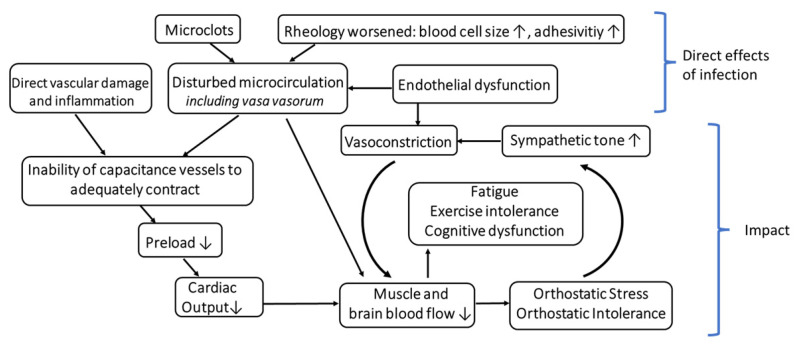
Hypothetical Role of the Capacitance Vessels and their Vasa Vasorum in Orthostatic Intolerance in the Post-COVID-19 Syndrome.

Altogether, the possibility should be considered that the vascular disturbance, which is undoubtedly present in Long COVID, does primarily cause OI and that autonomic dysfunction is the consequence thereof, which then contributes to worsening and maintaining of OD. Figure 1 shows the vicious cycle that could arise from these disturbances. From an epistemological point of view, it makes more sense starting the search or analysis of the potential causes of OI from the proven (micro)vascular disturbance than to assume a new and still vague concept like neuroinflammation.

How does the vascular disturbance of the capacitance system evolve over time? Assuming that a functional disturbance of capacitance vessels occurs in Long COVID to cause OI, as we assume here, the question is how this disturbance could evolve chronically when ME/CFS after COVID-19 is fully established. OI was already found to be highly prevalent in ME/CFS before the COVID-19 pandemic. Are the pathomechanisms causing OI the same in ME/CFS and Long COVID? Does the COVID-19-induced vascular disturbance, i.e., the presumed inability of the vascular media of the capacitance vessels to adequately contract, persist over time? As mentioned above, autonomic dysfunction, which, in our view, evolves secondary to the vascular disturbance and which is further enhanced by ME/CFS [48], indeed has the potential to contribute to the chronification of OI. A further finding in ME/CFS from investigations before COVID-19 is that hearts are smaller [50,51,52]. This may be due to the effect of a chronically low cardiac preload that is favored both by chronic hypovolemia and a disturbed capacitance vessel system unable to adequately fill the heart, which causes cardiac hypotrophy in the long run. Small hearts, in turn, may be another mechanism to contribute to the fixing of OI and maladaptation to exercise (exercise intolerance).

The assumed inability of the capacitance vessels to adequately contract during OR as a consequence of a microcirculatory disturbance of the vasa vasorum in Long COVID is not the only vascular pathomechanism potentially causing OD, as already mentioned above. The opposite disturbance, a rigid and perhaps shrunk capacitance vascular system, would probably also result in OD and maladaptation to exercise, as explained. It is worthwhile considering whether the latter is present or evolves in long-lasting ME/CFS as an alternative explanation for OI. The finding of small hearts could also suggest shrinkage of the cardiovascular low-pressure system. High sympathetic tone, low cardiac preload and hypovolemia are present in ME/CFS [51,52,53,54,55,56,57,58,59], which means that the diameter of these large vessels could be chronically smaller than normal. Does this also affect the structure of the blood vessels, which could mean fixation and shrinkage in a narrowed position similar to what we assume for the development of small hearts? Vessels are prone to long-term morphological changes, as known from chronic cardiovascular diseases like hypertension.

## 3. Concluding Remarks

Orthostatic intolerance observed in patients following infection with the COVID-19 virus and the development of Long COVID syndrome is induced by a pathologic response of the vasa vasorum of the capacitance vessels, leading to impaired vessel function. Dysfunction of the vasa vasorum includes endothelial dysfunction, microthrombus formation and disturbance of the rheological parameters of the blood cells. If the assumption holds true that the primary etiology of OI is a consequence of this microcirculatory dysfunction in contrast to the autonomic dysfunction, this would lead, in turn, to more specific treatment options and a potentially improved therapy outcome for the patient. Our intention is that instead of an a priori fixation on autonomic dysfunction, the dysfunction of capacitance vessels is taken into consideration as a potential primary cause of OI in Long COVID. Our assumptions for an altered capacitance vessel system explaining OD in Long COVID and ME/CFS are possibly amenable to experimental verification by comparing the diameters or volumes of large capacitance vessels in the recumbent and upright position in Long COVID and ME/CFS patients versus healthy controls.

## Data Availability

Not applicable.

## References

[1] Wong T.L., Weitzer D.J. (2021). Long Covid and Myalgic Encephalomyelitis/Chronic Fatigue Syndrome (ME/CFS)—A Systemic Review and Comparison of Clinical Presentation and Symptomatology. Medicina.

[2] Petracek L.S., Suskauer S.J., Vickers R.F., Patel N.R., Violand R.L., Swope R.L., Rowe P.C. (2021). Adolescent and Young Adult ME/CFS After Confirmed or Probable COVID-19. Front. Med..

[3] Jason L.A., Islam M.F., Conroy K., Cotler J., Torres C., Johnson M., Mabie B. (2021). COVID-19 symptoms over time: Comparing long-haulers to ME/CFS. Fatigue Biomed. Health Behav..

[4] Poenaru S., Abdallah S.J., Corrales-Medina V., Cowan J. (2021). COVID-19 and post-infectious myalgic encephalomyelitis/chronic fatigue syndrome: A narrative review. Ther. Adv. Infect. Dis..

[5] Sudre C.H., Murray B., Varsavsky T., Graham M.S., Penfold R.S., Bowyer R.C., Pujol J.C., Klaser K., Antonelli M., Canas L.S. (2021). Attributes and predictors of long COVID. Nat. Med..

[6] Mantovani E.A.O., Mariotto S., Gabbiani D., Dorelli G., Bozzetti S., Federico A., Zanzoni S., Girelli D., Crisafulli E., Ferrari S. (2021). Chronic fatigue syndrome: An emerging sequela in COVID-19 survivors?. J. Neurovirol..

[7] Freitag H., Szklarski M., Lorenz S., Sotzny F., Bauer S., Philippe A., Kedor C., Grabowski P., Lange T., Riemekasten G. (2021). Autoantibodies to Vasoregulative G-Protein-Coupled Receptors Correlate with Symptom Severity, Autonomic Dysfunction and Disability in Myalgic Encephalomyelitis/Chronic Fatigue Syndrome. J. Clin. Med..

[8] Kedor C., Freitag H., Meyer-Arndt L., Wittke K., Zoller T., Steinbeis F., Haffke M., Rudolf G., Heidecker B., Volk H.D. (2021). Chronic COVID-19 Syndrome and Chronic Fatigue Syndrome (ME/CFS) following the first pandemic wave in Germany—A first analysis of a prospective observational study. medRxiv.

[9] van Campen C.M.C., Rowe P.C., Visser F.C. (2021). Orthostatic Symptoms and Reductions in Cerebral Blood Flow in Long-Haul COVID-19 Patients: Similarities with Myalgic Encephalomyelitis/Chronic Fatigue Syndrome. Medicina.

[10] Carfì A., Bernabei R., Landi F. (2020). Persistent Symptoms in Patients After Acute COVID-19. JAMA.

[11] Davis H.E., Assaf G.S., McCorkell L., Wei H., Low R.J., Re’em Y., Redfield S., Austin J.P., Akrami A. (2021). Characterizing long COVID in an international cohort: 7 months of symptoms and their impact. eClinicalMedicine.

[12] Eldokla A.M., Ali S.T. (2022). Autonomic function testing in long-COVID syndrome patients with orthostatic intolerance. Auton. Neurosci..

[13] Kanjwal K., Jamal S., Kichloo A., Grubb B.P. (2020). New-onset Postural Orthostatic Tachycardia Syndrome following Coronavirus Disease 2019 Infection. J. Innov. Card. Rhythm. Manag..

[14] Novak P. (2020). Post COVID-19 syndrome associated with orthostatic cerebral hypoperfusion syndrome, small fiber neuropathy and benefit of immunotherapy: A case report. eNeurologicalSci.

[15] Carruthers B.M., van de Sande M.I., De Meirleir K.L., Klimas N.G., Broderick G., Mitchell T., Staines D., Powles A.C.P., Speight N., Vallings R. (2011). Myalgic Encephalomyelitis/Chronic Fatigue Syndrome. J. Chronic Fatigue Syndr..

[16] Roma M., Marden C.L., Flaherty M.A.K., Jasion S.E., Cranston E.M., Rowe P.C. (2019). Impaired Health-Related Quality of Life in Adolescent Myalgic Encephalomyelitis/Chronic Fatigue Syndrome: The Impact of Core Symptoms. Front. Pediatr..

[17] van Campen C.M., Verheugt F.W.A., Rowe P.C., Visser F.C. (2020). Cerebral blood flow is reduced in ME/CFS during head-up tilt testing even in the absence of hypotension or tachycardia: A quantitative, controlled study using Doppler echography. Clin. Neurophysiol. Pract..

[18] Novak P., Mukerji S.S., Alabsi H.S., Systrom D., Marciano S.P., Felsenstein D., Mullally W.J., Pilgrim D.M. (2021). Multisystem Involvement in Post-Acute Sequelae of Coronavirus Disease 19. Ann. Neurol..

[19] VanElzakker M.B., Brumfield S.A., Lara Mejia P.S. (2018). Neuroinflammation and Cytokines in Myalgic Encephalomyeli-tis/Chronic Fatigue Syndrome (ME/CFS): A Critical Review of Research Methods. Front Neurol..

[20] Jamal S.M., Landers D.B., Hollenberg S.M., Turi Z.G., Glotzer T.V., Tancredi J., Parrillo J.E. (2022). Prospective Evaluation of Autonomic Dysfunction in Post-Acute Sequela of COVID-19. J. Am. Coll. Cardiol..

[21] Acanfora D., Nolano M., Acanfora C., Colella C., Provitera V., Caporaso G., Rodolico G.R., Bortone A.S., Galasso G., Casucci G. (2022). Impaired Vagal Activity in Long-COVID-19 Patients. Viruses.

[22] Soliński M., Pawlak A., Petelczyc M., Buchner T., Aftyka J., Gil R., Król Z.J., Żebrowski J.J. (2022). Heart rate variability comparison between young males after 4–6 weeks from the end of SARS-CoV-2 infection and controls. Sci. Rep..

[23] Marques K.C., Costa Silva C., da Silva Trindade S., de Souza Santos M.C., Barbosa Rocha R.S., da Costa Vasconcelos P.F., Simões Quaresma J.A., Magno Falcão L.F. (2022). Reduction of Cardiac Autonomic Modulation and Increased Sympathetic Activity by Heart Rate Varia-bility in Patients with Long Covid. Front. Cardiovasc. Med..

[24] Jimeno-Almazán A., Pallarés J.G., Buendía-Romero A., Martínez-Cava A., Courel-Ibáñez J. (2021). Chronotropic Incompetence in Non-Hospitalized Patients with Post-COVID-19 Syndrome. J. Clin. Med..

[25] Barizien N., Le Guen M., Russel S., Touche P., Huang F., Vallée A. (2021). Clinical characterization of dysautonomia in long COVID-19 patients. Sci. Rep..

[26] Miwa K. (2017). Down-regulation of renin-aldosterone and antidiuretic hormone systems in patients with myalgic encephalomy-elitis/chronic fatigue syndrome. J. Cardiol..

[27] Wirth K., Scheibenbogen C. (2020). A Unifying Hypothesis of the Pathophysiology of Myalgic Encephalomyelitis/Chronic Fatigue Syndrome (ME/CFS): Recognitions from the finding of autoantibodies against ß2-adrenergic receptors. Autoimmun. Rev..

[28] Savastano M.C., Santoro L., Crincoli E., Fossataro C., Gambini G., Savastano A., De Vico U., Santoliquido A., Nesci A., Landi F. (2022). Radial Peripapillary Capillary Plexus Perfusion and Endothelial Dysfunction in Early Post-SARS-CoV-2 Infection. Vision.

[29] Charfeddine S., Ibn Hadj Amor H., Jdidi J., Torjmen S., Kraiem S., Hammami R., Bahloul A., Kallel N., Moussa N., Touil I. (2021). Long Covid 19 Syndrome: Is It Related to Microcirculation and Endothelial Dysfunction? Insights From TUN-EndCOV Study. Front. Cardiovasc. Med..

[30] Mejia-Renteria H., Travieso A., Sagir A., Martínez-Gómez E., Carrascosa-Granada A., Toya T., Núñez-Gil I.J., Estrada V., Lerman A., Escaned J. (2021). In-vivo evidence of systemic endothelial vascular dysfunction in COVID-19. Int. J. Cardiol..

[31] Sang C.J., Burkett A., Heindl B., Litovsky S.H., Prabhu S.D., Benson P.V., Rajapreyar I. (2021). Cardiac pathology in COVID-19: A single center autopsy experience. Cardiovasc. Pathol..

[32] Pretorius E., Vlok M., Venter C., Bezuidenhout J.A., Laubscher G.J., Steenkamp J., Kell D.B. (2021). Persistent clotting protein pathology in Long Covid/Post-Acute Sequelae of COVID-19 (PASC) is accom-panied by increased levels of antiplasmin. Cardiovasc. Diabetol..

[33] Saha A.K., Schmidt B.R., Wilhelmy J., Nguyen V., Abugherir A., Do J.K., Nemat-Gorgani M., Davis R.W., Ramasubramanian A.K. (2019). Red blood cell deformability is diminished in patients with Chronic Fatigue Syndrome. Clin. Hemorheol. Microcirc..

[34] Zhu Y., Chen X., Liu X. (2022). NETosis and Neutrophil Extracellular Traps in COVID-19: Immunothrombosis and Beyond. Front. Immunol..

[35] Vasuri F., Ciavarella C., Collura S., Mascoli C., Valente S., Degiovanni A., Gargiulo M., Capri M., Pasquinelli G. (2021). Adventitial Microcirculation Is a Major Target of SARS-CoV-2-Mediated Vascular Inflammation. Biomolecules.

[36] Boyle E.C., Haverich A. (2020). Microvasculature dysfunction as the common thread between atherosclerosis, Kawasaki disease, and severe acute respiratory syndrome coronavirus 2 (SARS-CoV-2)-associated multi-system inflammatory syndrome in children. Eur. J. Cardiothorac. Surg..

[37] Daisley H., Rampersad A., Daisley M., Ramdin A., Acco O., Narinesingh F., Humphrey O., James E. (2021). The vasa vasorum of the large pulmonary vessels are involved in COVID-19. Autops. Case Rep..

[38] Faa G., Gerosa C., Fanni D., Barcellona D., Cerrone G., Orrù G., Scano A., Marongiu F., Suri J.S., Demontis R. (2021). Aortic vulnerability to COVID-19: Is the microvasculature of vasa vasorum a key factor? A case report and a review of the literature. Eur. Rev. Med. Pharmacol. Sci..

[39] Sollini M., Ciccarelli M., Cecconi M., Aghemo A., Morelli P., Gelardi F., Chiti A. (2020). Vasculitis changes in COVID-19 survivors with persistent symptoms: An [18F]FDG-PET/CT study. Eur. J. Nucl. Med..

[40] Heistad D.D., Armstrong M.L., Amundsen S. (1986). Blood flow through vasa vasorum in arteries and veins: Effects of luminal PO_2_. Am. J. Physiol. Circ. Physiol..

[41] Brook W. (1977). Vasa Vasorum of Veins in Dog and Man. Angiology.

[42] Vlachopoulos C., Terentes-Printzios D., Katsaounou P., Solomou E., Gardikioti V., Exarchos D., Economou D., Christopoulou G., Kalkinis A.-D., Kafouris P. (2022). Time-related aortic inflammatory response, as assessed with 18F-FDG PET/CT, in patients hospitalized with severely or critical COVID-19: The COVAIR study. J. Nucl. Cardiol..

[43] Wirth K.J., Scheibenbogen C. (2021). Pathophysiology of skeletal muscle disturbances in Myalgic Encephalomyelitis/Chronic Fatigue Syndrome (ME/CFS). J. Transl. Med..

[44] Wirth K.J., Scheibenbogen C. (2022). Dyspnea in Post-COVID Syndrome following Mild Acute COVID-19 Infections: Potential Causes and Consequences for a Therapeutic Approach. Medicina.

[45] Hejbøl E.K., Harbo T., Agergaard J., Madsen L.B., Pedersen T.H., Østergaard L.J., Andersen H., Schrøder H.D., Tankisi H. (2022). Myopathy as a cause of fatigue in long-term post-COVID-19 symptoms: Evidence of skeletal muscle his-topathology. Eur. J. Neurol..

[46] Campen C., Rowe P., Visser F. (2020). Reductions in Cerebral Blood Flow Can Be Provoked by Sitting in Severe Myalgic Encephalomyelitis/Chronic Fatigue Syndrome Patients. Healthcare.

[47] Van Campen C.L.M.C., Rowe P.C., Visser F.C. (2020). Cerebral Blood Flow Is Reduced in Severe Myalgic Encephalomyelitis/Chronic Fatigue Syndrome Patients During Mild Orthostatic Stress Testing: An Exploratory Study at 20 Degrees of Head-Up Tilt Testing. Healthcare.

[48] Wirth K.J., Scheibenbogen C., Paul F. (2021). An attempt to explain the neurological symptoms of Myalgic Encephalomyeli-tis/Chronic Fatigue Syndrome. J. Transl. Med..

[49] Szewczykowski C., Mardin C., Lucio M., Wallukat G., Hoffmanns J., Schröder T., Raith F., Rogge L., Heltmann F., Moritz M. (2022). Long COVID: Association of Functional Autoantibodies against G-Protein-Coupled Receptors with an Impaired Retinal Microcirculation. Int. J. Mol. Sci..

[50] Miwa K., Fujita M. (2009). Cardiovascular Dysfunction with Low Cardiac Output Due to a Small Heart in Patients with Chronic Fatigue Syndrome. Intern. Med..

[51] Hollingsworth K.G., Hodgson T., MacGowan G.A., Blamire A.M., Newton J.L. (2011). Impaired cardiac function in chronic fatigue syndrome measured using magnetic resonance cardiac tagging. J. Intern. Med..

[52] Miwa K. (2015). Cardiac dysfunction and orthostatic intolerance in patients with myalgic encephalomyelitis and a small left ventricle. Heart Vessel..

[53] Newton J.L., Finkelmeyer A., Petrides G., Frith J., Hodgson T., Maclachlan L., MacGowan G., Blamire A.M. (2016). Reduced cardiac volumes in chronic fatigue syndrome associate with plasma volume but not length of disease: A cohort study. Open Hear..

[54] Hurwitz B.E., Coryell V.T., Parker M., Martin P., Laperriere A., Klimas N.G., Sfakianakis G.N., Bilsker M.S. (2009). Chronic fatigue syndrome: Illness severity, sedentary lifestyle, blood volume and evidence of di-minished cardiac function. Clin. Sci..

[55] De Becker P., Dendale P., De Meirleir K., Campine I., Vandenborne K., Hagers Y. (1998). Autonomic testing in patients with chronic fatigue syndrome. Am. J. Med..

[56] Wyller V.B., Saul J.P., Walløe L., Thaulow E. (2007). Sympathetic cardiovascular control during orthostatic stress and isometric exercise in adolescent chronic fatigue syndrome. Eur. J. Appl. Physiol..

[57] Rimes K.A., Lievesley K., Chalder T. (2017). Stress vulnerability in adolescents with chronic fatigue syndrome: Experimental study investigating heart rate variability and skin conductance responses. J. Child Psychol. Psychiatry.

[58] Burton A.R., Rahman K., Kadota Y., Lloyd A., Vollmer-Conna U. (2010). Reduced heart rate variability predicts poor sleep quality in a case-control study of chronic fatigue syn-drome. Exp. Brain Res..

[59] Joseph P., Arevalo C., Oliveira R.K.F., Faria-Urbina M., Felsenstein D., Oaklander A.L., Systrom D.M. (2021). Insights From Invasive Cardiopulmonary Exercise Testing of Patients with Myalgic Encephalomyeli-tis/Chronic Fatigue Syndrome. Chest.

